# Combustion Synthesis of Porous TiC/Ti Composite by a Self-Propagating Mode

**DOI:** 10.3390/ma3073939

**Published:** 2010-07-06

**Authors:** Makoto Kobashi, Daishi Ichioka, Naoyuki Kanetake

**Affiliations:** 1 Graduate School of Engineering, Nagoya University, 1 Furo-cho, Chikusa-ku, Nagoya, Aichi 464-8603, Japan; E-Mail: kanetake@numse.nagoya-u.ac.jp (N.K.); 2 Graduate student, Nagoya University, 1 Furo-cho, Chikusa-ku, Nagoya, Aichi 464-8603, Japan

**Keywords:** self-propagating high-temperature synthesis (SHS), porous material, cell material, biomaterials, metal matrix composites (MMCs), combustion temperature

## Abstract

Porous titanium carbide (TiC) and TiC/Ti composites were synthesized by self-propagating high-temperature synthesis (SHS). Titanium and carbon powders were blended by various Ti/C blending ratios. The heat of reaction between titanium and carbon was high enough to induce the self-sustaining reaction of TiC formation on condition that some processing parameters (Ti/C ratio and porosity of the precursor) were appropriately selected. When the Ti/C blending ratio was high, the excess amount of titanium absorbed the heat of reaction. Consequently, the heated zone was not heated up to the ignition temperature. On the other hand, when the Ti/C ratio was low, high thermal conductivity of the precursor prevented an ignition of the heated side of precursors. The pore morphology was controlled by changing the Ti/C ratio and the preheat temperature.

## 1. Introduction

Titanium alloys and titanium carbide (TiC) ceramics have attracted a great deal of attention due to their excellent mechanical properties, good corrosion resistance and biocompatibility [1,2,3]. Porous metals and ceramics have been studied by many researchers for medical purposes (artificial hard-tissue replacements, for example), and it is well-known that porous materials are suitable for surgical implants because the pores permit bone ingress and give a firm bond between the implants and the human bone [4]. The pore size is one of the most important characteristics for the porous implant materials, and an optimum range of the pore size was reported roughly from 200 μm to 500 μm [1]. The pore morphology and porosity are also important characteristics, which regulate physical properties of the porous materials. Apart from the bio-oriented applications, the porous Ti alloys and TiC ceramic are applicable to tribological purposes and to filtering materials under extremely severe environments (high-temperature or highly corrosive environments).

**Figure 1:**
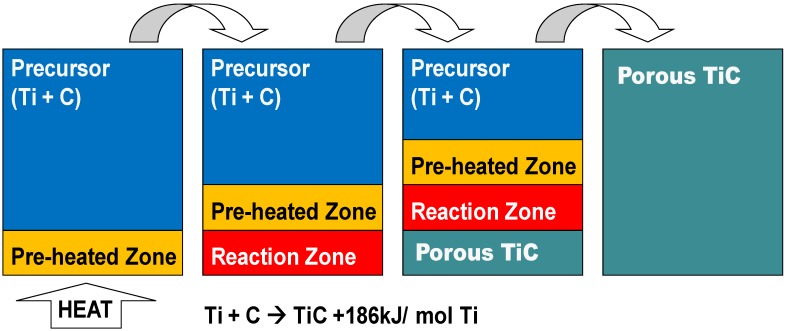
A brief outline of the SHS process (TiC formation).

Porous titanium alloys and TiC ceramics are generally fabricated by a sintering process, but a disadvantage is that this process consumes large amounts of energy. In contrast to the conventional sintering process, self-propagating high-temperature synthesis (SHS) is a cost-effective process that makes use of a large amount of heat of reaction. Figure 1 shows a brief outline of the SHS process of the TiC formation from a precursor consisting of elemental titanium and carbon powders. In the SHS process, only a part of the precursor needs to be heated. Once the temperature at the heated zone in Figure 1 reaches to the ignition temperature (T_ig_) [5], then a TiC formation occurs (reaction zone in Figure 1), and the heat of reaction shown below is released

Ti + C ➔ TiC + Q Q = 186kJ/ mol Ti
(1)

This heat of reaction raises the temperature of the neighboring zone and induces the TiC formation again. Thus the reaction propagates to the other end of the specimen spontaneously. Since the SHS process uses the heat of reaction, only a small amount of energy is required to synthesize ceramics or intermetallics [6,7,8]. The following three factors are the typical characteristics of the SHS process.

● simple processing (no special equipment is required);
● short-time processing (reaction completes in several seconds);
● synthesis of porous materials.

The SHS process is regarded as a prominent and cost-effective processing method to produce porous materials. The purpose of this work is to synthesize porous TiC/Ti composites with pore sizes below 500 μm by the SHS process. In this paper, the following factors are discussed;

● effects of the blending ratio of starting materials (Ti/C) on the SHS reaction and pore morphology
● the thermodynamic control of the pore size by preheating the precursor

## 2. Experimental Procedure

**Figure 2:**
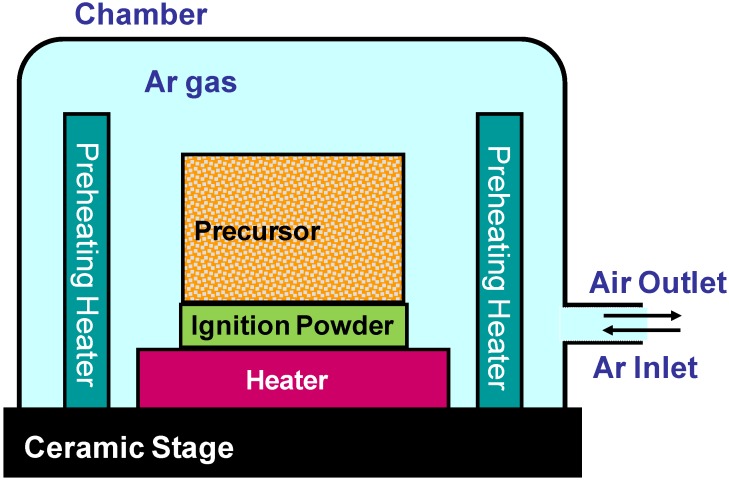
Schematic illustration of the experimental apparatus.

Starting materials used in this experiment were titanium powder (99.4% pure, size: < 45 μm) and carbon powder (99.9% pure, size: < 5 μm). First of all, the starting powders were blended by molar blending ratios (Ti/C) ranging from 1.33 to 2.99. The blended powder was then compressed by different compacting pressures (17–165 MPa) to prepare precursors (cylindrical shape: 15 mm in diameter, 10 mm in height). The precursor was placed in a furnace as shown in Figure 2. The precursor was located on an ignition powder compact (15 mm in diameter, 2 mm in height, relative density: 0.85) in a vacuum chamber. As for the ignition powder, [3Al + 3Ti + B_4_C] blended powder was used [9]. This ignition powder compact reacts at 933 K (melting point of aluminum), and generates a large heat of reaction (254 kJ/mole Ti) and ignited the reaction of the precursor. The chamber was evacuated by a rotary pump and then backfilled with Ar gas. In some experiments, the precursor was preheated (473 K) prior to igniting the SHS reaction. After the reaction, the cross section of the specimen was observed by optical microscopy (OM) and scanning electron microscopy (SEM) and analyzed by an X-ray diffraction (XRD) method.

## 3. Results

### 3.1. Self-Propagation of the Reaction

Table 1 shows the possibility of the ignition and self-propagation of the reaction with various Ti/C blending ratios (from 1.33 to 2.99). “*Ignition*” means that the formation of TiC was confirmed at the bottom of the precursor. “*Self-propagation*” means that the reaction propagated from the heated side to the non-heated side. The compacting pressure for preparing the precursor was fixed to 55 MPa (porosity of the precursor: 35%). The SHS reaction successfully occurred only when the Ti/C ratio was 1.88 and 2.33. The formation of TiC was not observed even at the bottom of the precursor when the Ti/C ratio was 1.33, 1.59, meaning that the precursor was not ignited at all. Only the bottom of the precursor was reacted when Ti/C ratio was 2.99, meaning that the reaction did not propagate. This result suggests that there is an adequate range in the Ti/C ratio to induce the self-propagating reaction. The following two properties are important parameters for the possibility of the SHS reaction.

● Thermal Conductivity: the temperature of the heated side of the precursor should be raised to the ignition temperature to induce the self-sustaining reaction. This condition is sometimes difficult to achieve when the thermal conductivity of the precursor is high.
● Heat of Reaction: when a material, which is not involved in the reaction, is blended in the precursor, such a material absorbs the heat of reaction. As a result of the absorption, the temperature of the precursor during the SHS reaction (Combustion Temperature: *Tc*) is reduced, and the heated zone ca not reach the ignition temperature.

Since thermal conductivity of carbon is much higher than that of titanium (C: 900–2000 W/m･K, Ti:21.9 W/m･K), the precursors with low Ti/C ratios (1.33 and 1.59) conduct more heat than other specimens. Judging from the fact that the self-propagation occurred when Ti/C ratio was 1.88 or 2.33, it is no doubt that the self-propagation of the specimens with Ti/C ratios of 1.33 and 1.59 takes place once the reaction at the heated side is ignited. However, in this experiment, the heat flux from the ignition powder was not sufficient enough to raise the temperature of the precursor up to the ignition temperature due to high thermal conductivity. Then, the ignition did not occur when Ti/C ratios were 1.33 and 1.59. On the other hand, high Ti/C ratio (2.99) produced an excessive amount of titanium, which could not be involved in the reaction. This excess titanium phase absorbed the heat of reaction and reduced the combustion temperature, which prevented a sufficient increase in the temperature of the heated zone to the ignition temperature.

Table 2 shows the possibility of the SHS reaction of precursors with various porosities. The porosity was varied from 13 to 70% by controlling the powder compacting pressures. The Ti/C ratio was fixed to 2.33 in this experiment. The SHS reaction occurred when the porosity of the precursor was higher than 35%, whereas the specimens with 13 and 24% porosities did not show the SHS reaction. The precursors with low porosity exhibits relatively higher thermal conductivity, and this leads to the difficulty of the SHS reaction.

**Table 1:** Effect of the Ti/C ratio on the possibility of the SHS reaction.

TiC ratio	Compacting Pressure (MPa)	Theoretical V_f_ of TiC	Possibility of Ignition	Possibility of Self-propagation
2.99	55 (Porosity: 35%)	40%	Yes	No
2.33		50%	Yes	Yes
1.88		60%	Yes	Yes
1.59		70%	No	-
1.33		80%	No	-

**Table 2:** Effect of porosity of the precursor on the possibility of the SHS reaction.

TiC ratio	Compacting Pressure (MPa)	Porosity	Possibility of Ignition	Possibility of Self-propagation
2.33 (V_f_ of TiC: 50%)	17	50	Yes	Yes
	28	45	Yes	Yes
	55	35	Yes	Yes
	110	24	No	-
	165	13	No	-

### 3.2. Ti/C Blending Ratio

**Figure 3:**
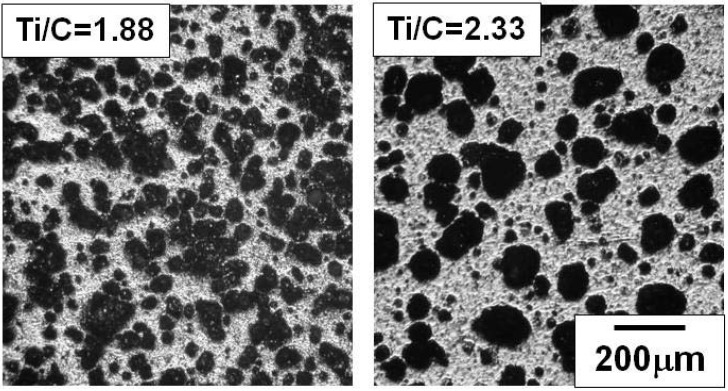
Macroscopic view of combustion synthesized specimens (Ti/C molar ratio = 1.88 and 2.33).

**Figure 4:**
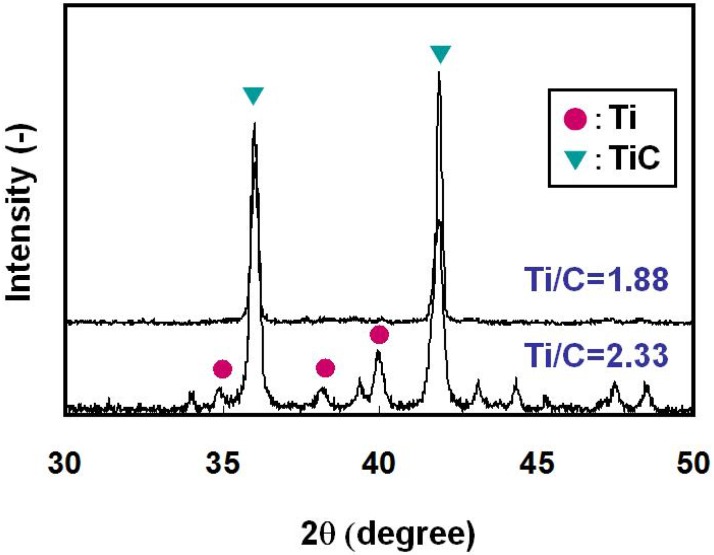
XRD patterns of combustion synthesized specimens (Ti/C ratio = 1.88 and 2.33).

**Figure 5:**
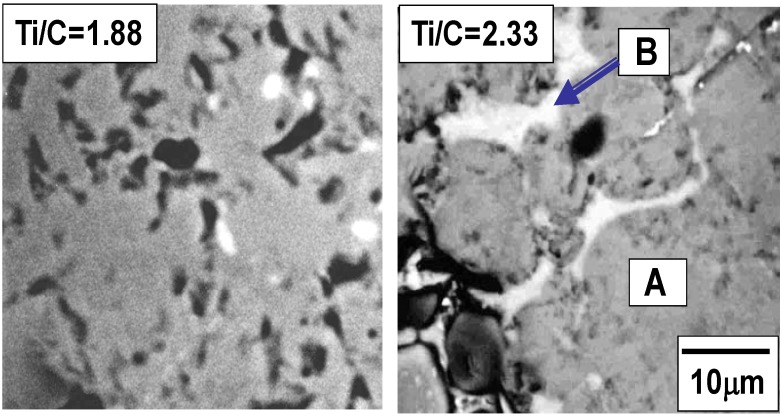
SEM image of combustion synthesized specimens (Ti/C molar ratio = 1.88 and 2.33). A and B in the right panel are the points where EDX analysis was carried out.

**Figure 6:**
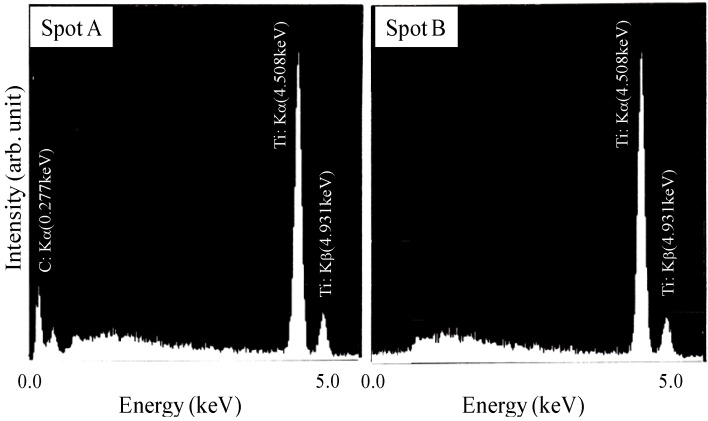
Typical characteristic X-ray spectra detected at bright (spot A) and dark(spot B) regions in Figure 5.

Figure 3 shows the cross section of the specimens after the SHS reaction with the Ti/C ratios of 1.88 and 2.33. As already indicated in the former section, the reaction propagated throughout the specimen successfully with these Ti/C ratios. The bright region and the dark region in Figure 3 are the TiC/Ti composite and pores, respectively. Pores with average diameter of 50μm (Ti/C = 1.88) and 100 μm (Ti/C = 2.33) are identified, which implies that the pore size was affected by the Ti/C blending ratio. Figure 4 shows the XRD patterns of these specimens. When the Ti/C ratio was 2.33, the X-ray diffraction peaks of both titanium and TiC were clearly detected at appropriate angular positions, whereas the diffraction peaks of titanium were scarcely detected and only the TiC peaks were detected when the Ti/C ratio was 1.88. Figure 5 shows the microscopic cross sections of the two specimens observed by SEM. Figure 6 shows energy dispersive characteristic X-ray spectra detected from points A and B in figure 5. Judging from Figure 4 and Figure 6, both titanium and TiC were observed when the Ti/C ratio was 2.33, but only a minute quantity of titanium was observed when the Ti/C ratio was 1.88.

Titanium carbide has a cubic NaCl-type structure, which has a wide composition range between TiC_0.49_–TiC_0.95_ [10] and the composition of non-stoichiometric TiC_x_ can be predicted from the Ti-C phase diagram. The formation of monolithic TiC_0.53_ phase was expected when the Ti/C ratio was 1.88, and Figure 4 and Figure 5 proved the formation of nearly monolithic phase. When the Ti/C ratio was 2.33, the formation of TiC_0.49_ and the remaining titanium was expected from the phase diagram, and confirmed experimentally as already indicated in Figure 4 and Figure 5.

The remaining titanium played an important role in determining the pore morphology. The mechanisms of pore formation are considered as follows;

● original pores in the precursor remains after the SHS reaction
● diffusion of molten titanium into carbon generates pores where titanium powder was originally located.

If the combustion temperature exceeded the melting point of titanium, the fluidity of the specimen sharply increased by the presence of the liquid phase; this allowed the deformation and connection of pores, and also contributed to the growth of the pore size as shown in Figure 3.

### 3.3. Preheating the Precursor

**Figure 7:**
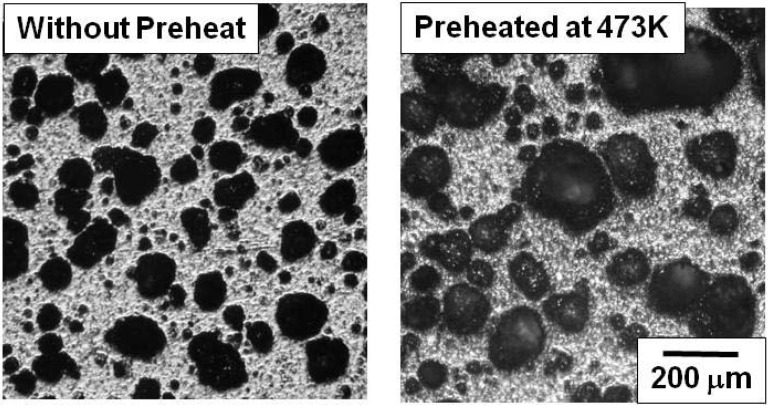
Macroscopic view of combustion synthesized specimens with and without preheating (preheating temperature = 473K, Ti/C ratio = 2.33).

Preheating the precursor prior to the SHS reaction was carried out and the effect of preheating on the pore morphology is discussed in this session. The role of preheating was to regulate the combustion temperature and to control the pore morphology. Figure 7 shows the cross section of the specimens (Ti/C = 2.33) without preheating and with preheating at 473 K. The precursors of both specimens were compressed by 55 MPa (porosity: 35%). The larger pores (200 μm) were formed by preheating the specimen. By preheating the precursor, the combustion temperature (T_c_) was raised. The combustion temperature is calculated thermodynamically by the following equation.

(2) $$\begin{array}{l} \Delta H_{f}\left(TiC\right)\;+\;\int_{T_{ig}}^{T_{c}}\left(C_{p}\left(TiC\right)\right)\;dT\\ \;+\;\left(\alpha -1\right)\cdot \left(\int_{T_{ig}}^{1939}C_{p}\left(Ti\right)\;dT\;+\;\Delta H_{melt}\left(Ti\right)+\int_{1939}^{Tc}C_{p}\left(Ti\right)\;dT\right)\\ \;=\;0 \end{array}$$

where ΔH_f_ denotes the heat of reaction (J/mol), ΔH_melt_ denotes the latent heat of melting (J/mol), C_p_ denotes the specific heat (J/K･mol), T_0_ denotes the initial temperature (K), α denotes the molar Ti/C ratio.

**Figure 8:**
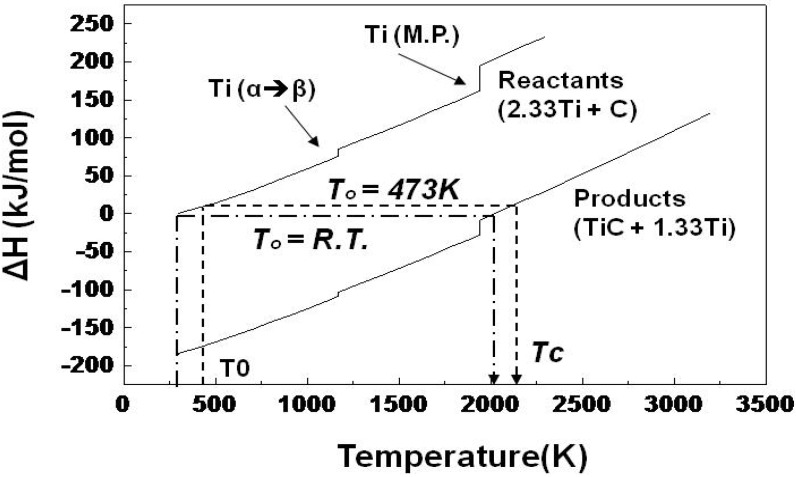
Enthalpy data of the Ti-C system (Ti/C = 2.33) for the calculation of the adiabatic combustion temperature.

Figure 8 shows the enthalpy data of the reactants (titanium and carbon) and the product (TiC) calculated form Eq. (2). According to the calculation, the combustion temperature is above the melting point of titanium, and it is also understood that the preheating effectively raises the combustion temperature. This means that fluidity of the liquid phase during the SHS reaction becomes higher by preheating the precursor, which promotes the deformation and connection (coarsening) of pores.

## 4. Conclusions

The porous TiC/Ti composite was fabricated by self-propagating high-temperature synthesis. The powder blending ratio, porosity of the precursor and preheating temperature were regarded as important processing parameters to control the SHS reaction and pore morphology. The results obtained by the present work are summarized as follows.

1) The heat of reaction between titanium and carbon was high enough to induce the SHS reaction on condition that the Ti/C blending ratio was appropriately selected (1.88 and 2.33). The lower Ti/C ratios (1.33 and 1.59) did not allow the ignition because the thermal conductivity of the precursor was too high to raise the heated side of the precursor to the ignition temperature. When the Ti/C ratio was high (2.99), the heat of reaction was absorbed by the excess amount of titanium. As a result, the heated zone was not heated up to the ignition temperature.
2) Porosity of the precursor should be higher than a critical value to reduce the thermal conductivity of the precursor.
3) The pore size could be varied from 50μm to 100μm by changing the Ti/C blending ratio from 1.88 to 2.33.
4) By preheating the precursor, the combustion temperature was raised and the liquid phase formation also increased, which afterwards attributed to the larger pore formation.
